# Supplementary material for: Proof-of-Concept: Antisense Oligonucleotide Mediated Skipping of Fibrillin-1 Exon 52
Source: Int J Mol Sci. 2021 Mar 27;22(7):3479. doi: 10.3390/ijms22073479 (PMC8037683; doi:10.3390/ijms22073479)
Supplement: Supplementary file 1 [file ijms-22-03479-s001.pdf]

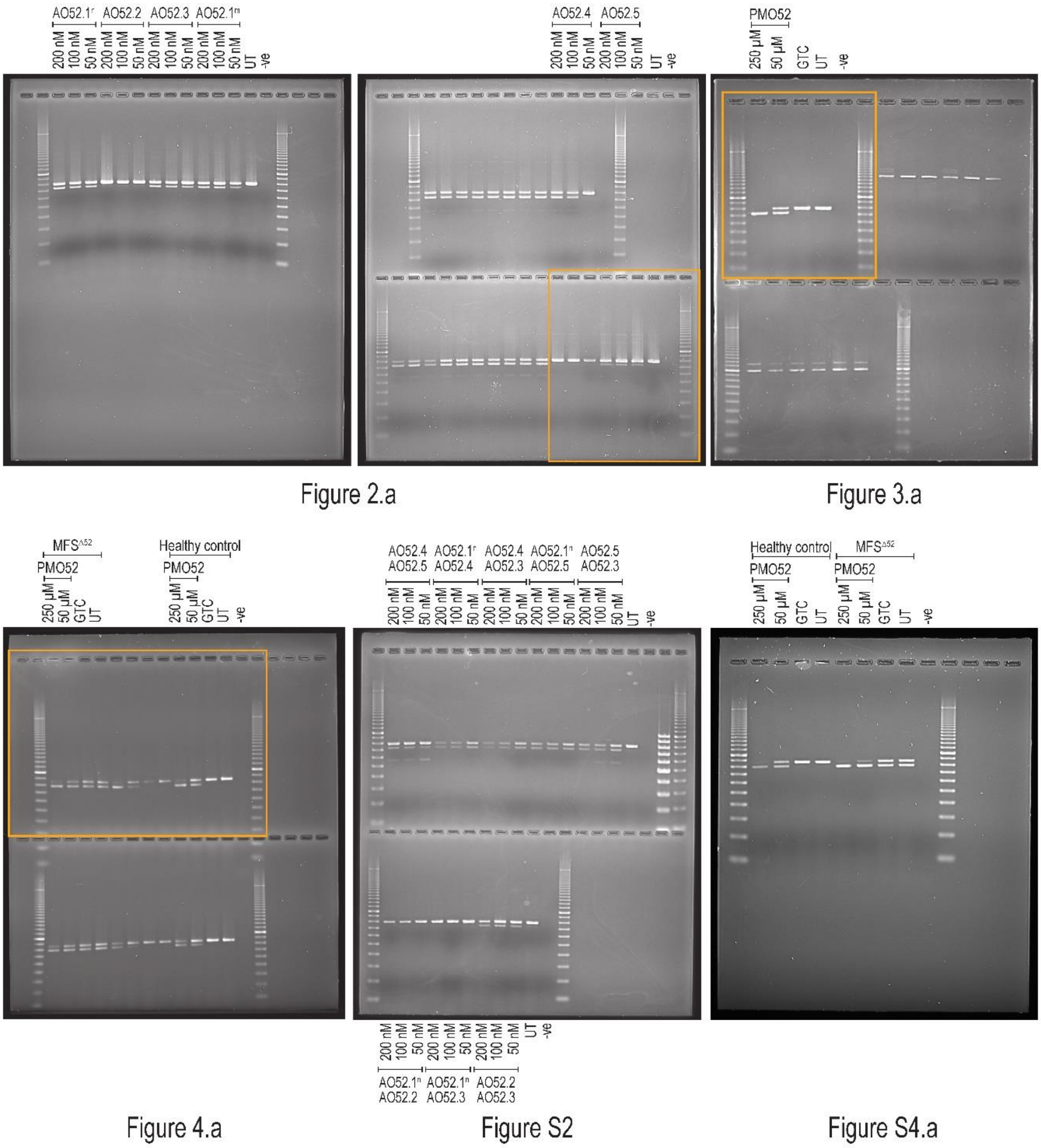

Figure S1. Full gel images for figures listed.

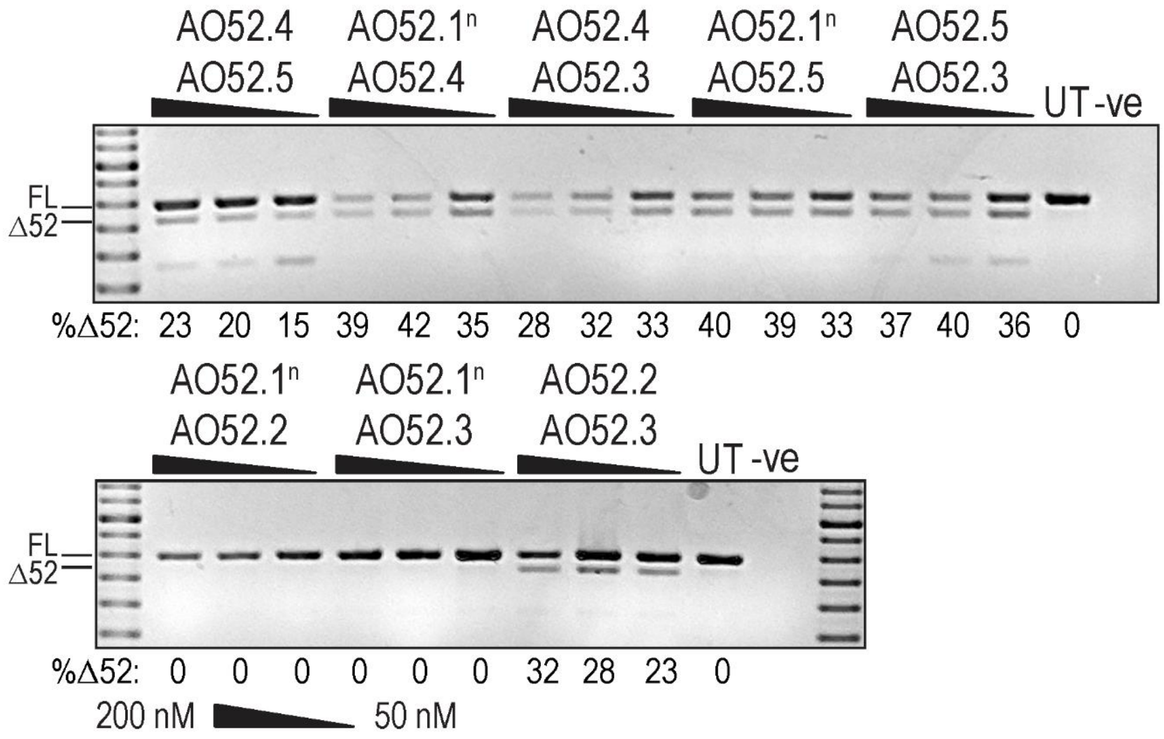

Figure S2. Evaluation of AO cocktails designed to induce FBN1 exon 52 skipping. Healthy control fibroblasts were transfected with AOs at three concentrations, 200, 100 and 50 nM. The values below each gel image indicate the percentage of exon 52 skipped (Δ52) transcripts in each sample. Ctrl: an unrelated sequence used as a sham treatment, UT: untreated control, ve: RT-PCR negative control. 100bp molecular marker used for size reference.

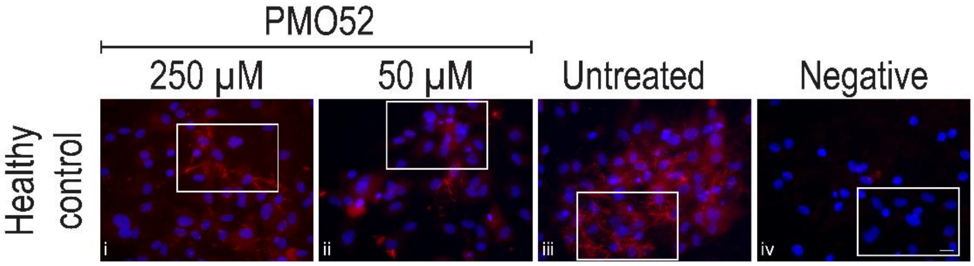

Figure 3.b

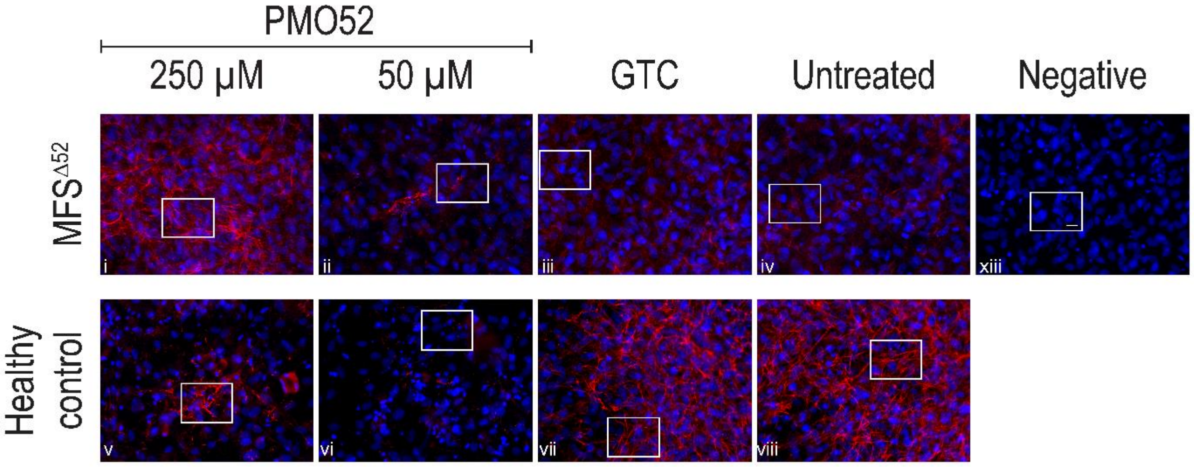

Figure 4.b

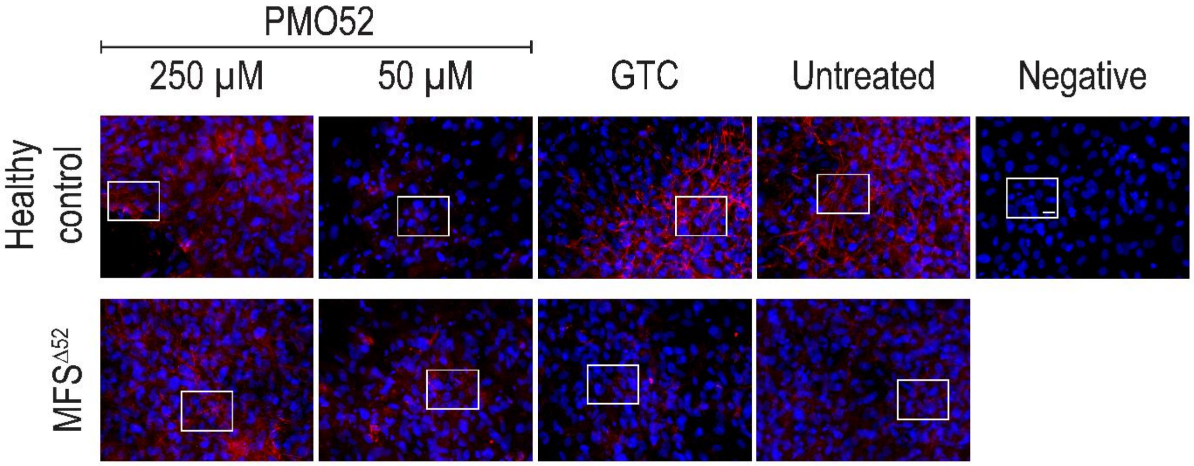

Figure S4.b

Figure S3. Full immunofluorescence staining images for figures listed.

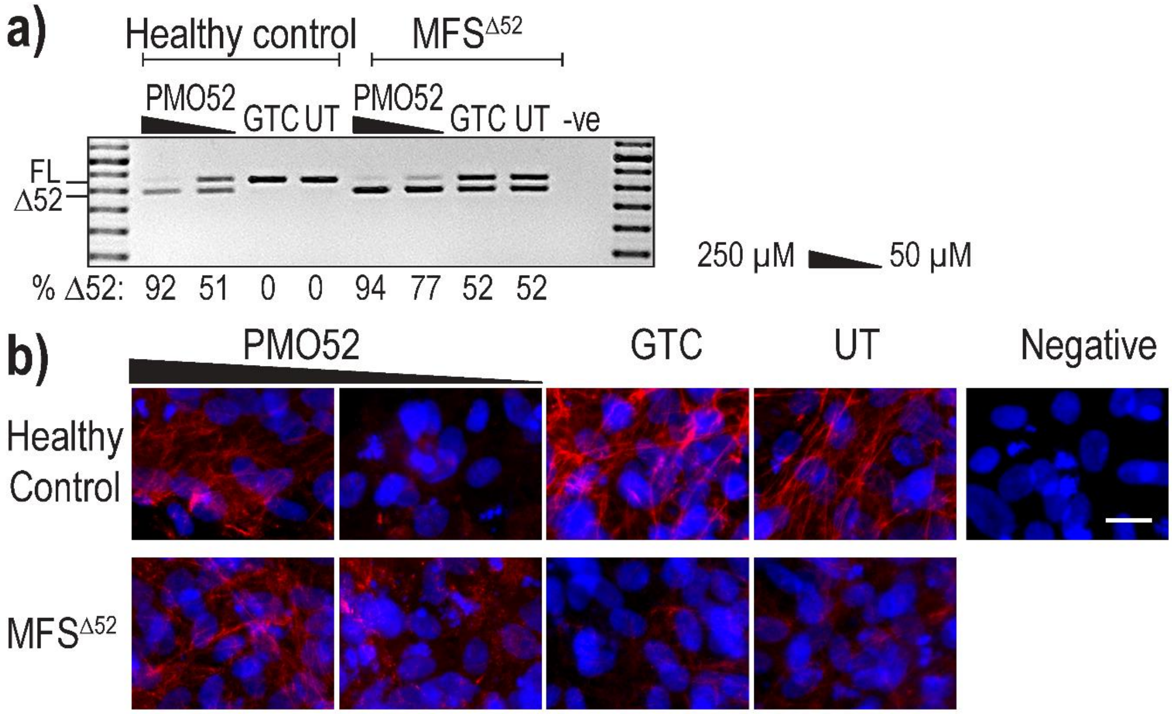

Figure S4. Additional evaluation of PMO52. Healthy control and MFSΔ52 fibroblasts were transfected with PMO52 (250 μM and 50 μM, GTC (250 μM) or left untreated. Cells were collected 72 hours post-transfection. (**a**) RT-PCR analysis of FBN1 exons 47-54 amplicons showing full-length (FL, 859bp) and exon 52-skipped (Δ52, 793bp) transcripts. The relative abundance (%) of Δ52 amplicons are shown below the gel image. GTC: Gene Tools control PMO, UT: untreated control, -ve: RT-PCR negative control, 100bp molecular marker used as a size reference. The gel was cropped for presentation. Full gel image is presented in Figure S1. (**b**) Representative images showing fibrillin-1 (red) and nuclei (blue). Negative: No primary antibody added. Scale bar =20 μm. The images were cropped for presentation. Full images are presented in Figure S3.
